# A Smartphone-Assisted Approach to Formaldehyde Detection Using Diethanolamine-Grafted Carbon Nanoparticles

**DOI:** 10.1021/acsomega.6c00964

**Published:** 2026-06-05

**Authors:** Rossella Santonocito, Lorenzo Russo, Victor Sebastian, Angelo Ferlazzo, Antonino Gulino, Manuel Petroselli, Roberta Ruffino, Giovanni Li Destri, Andrea Pappalardo, Nunzio Tuccitto, Alessia Cavallaro, Giuseppe Trusso Sfrazzetto

**Affiliations:** † Department of Chemical Sciences, 9298 University of Catania, viale A. Doria 6, Catania 95125, Italy; ‡ Instituto de Nanociencia y Materiales de Aragoń Aragón (INMA), 16765 CSIC-Universidad de Zaragoza, Campus Rio Ebro, Edificio I + D + I, C/Poeta Mariano Esquillor, s/n, Zaragoza 50018, Spain; § Department of Chemical and Environmental Engineering, Institute of Nanoscience and Materials of Aragon, Universidad de Zaragoza, Zaragoza 50018, Spain; ∥ Networking Research Center in Biomaterials, Bioengineering and Nanomedicine (CIBER-BBN), Instituto de Salud Carlos III, Madrid 28029, Spain; ⊥ Laboratorio de Microscopías Avanzadas, Univeridad de Zaragoza, Zaragoza 50018, Spain; # Department of Science and Technological Innovation, University of Eastern Piedmont “Amedeo Avogadro”, Viale Teresa Michel 11, Alessandria 15121, Italy; ¶ CSGI Consorzio Interuniversitario per Lo sviluppo Dei Sistemi a Grande Interfase, Via della Lastruccia 3, Firenze 50019, Italy

## Abstract

Formaldehyde (FA) is a well-known indoor pollutant and human carcinogen, making the development of field-deployable sensors for its monitoring crucial for public safety. Herein, we report a facile strategy to synthesize carbon nanoparticles functionalized with diethanolamine (CNPs-DEA) as FA sensors. The strategic surface functionalization dictates a specific spatial arrangement that is key to efficient and cooperative interaction with the analyte. In water solution, the probe exhibits a LOD of 46 ppb with a binding constant (log β) value of 4.31 ± 0.01. By immobilizing the CNPs on polyamide membranes, we engineered a solid-state strip test integrated with a 3D-printed smartphone readout platform. This setup delivers an ultralow detection threshold (1 ppb) for gaseous FA and, crucially, maintains robust performance under saturated humidity conditions, overcoming a major limitation of conventional carbon-based sensors. The practical utility of the device was validated by quantifying FA in commercial paint samples. Combining operational simplicity, low cost, and high sensitivity, this platform represents a robust early warning tool for air quality monitoring, bridging the gap between sophisticated laboratory instrumentation and accessible real-world applications.

## Graphical abstract

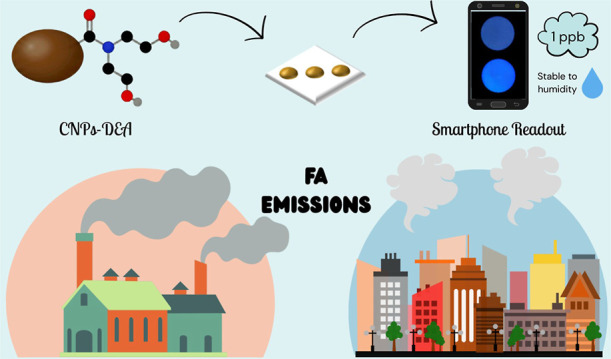

## 1 Introduction

Indoor air quality has become a major public health concern, with formaldehyde (FA) identified as one of the most critical volatile organic compounds (VOCs) in residential and occupational environments.^1^ Unlike outdoor environments, where FA concentrations are typically diluted by atmospheric dispersion, indoor spaces accumulate this pollutant due to continuous off-gassing from building materials, furniture, adhesives, and consumer products.^2^ Regulatory agencies worldwide have responded to this issue: the lowest threshold has been established by World Health Organization (WHO), that set a 30 min exposure limit of 0.1 mg m^–3^ (80 ppb) in indoor spaces.^3^ These restrictive guidelines reflect FA’s toxicological profile, as its chronic inhalation is associated with respiratory disfunction and eye irritation.^4^ Of greater concern is FA’s classification as carcinogenic to humans (Group 1) by the International Agency for Research on Cancer (IARC), with connections to lung cancer and leukemia.^5^ The reactive nature of FA as a carbonyl compound, combined with its high volatility and water solubility, makes it particularly challenging to monitor and control in real-world settings.^6^

Accurate and timely detection of FA is essential for exposure assessment and risk management. Traditional analytical methods, such as high-performance liquid chromatography (HPLC)^7^ or gas chromatography using mass spectrometry as detector (GC–MS),^8^ provide excellent sensitivity and selectivity, but their use is limited by high costs of the instruments, complex use that require qualified operators and sample preparation requirements that preclude point-of-need monitoring. Electrochemical sensors offer integrated alternatives with faster response times,^9^ yet their performance can be compromised by cross-sensitivity to other aldehydes and common atmospheric trace gases, such as NO_2_, O_3_ and CO, requiring frequent calibration and maintenance.^10^ Optical sensing approaches, particular those based on fluorescence modulation, have emerged as attractive solutions that balance sensitivity, selectivity and operational simplicity.^11,12^ However, molecular probes in solution can suffer from limited binding efficiency at trace concentration levels, highlighting the need for advanced sensing architectures.

In recent years, nanostructured materials have demonstrated extraordinary versatility across diverse fields, ranging from the development of advanced pressure sensors^13^ and antibacterial platforms^14^ to innovative strategies for cancer screening.^15^ In particular, they have revolutionized the field of chemical sensing by providing enhanced surface activity, increased binding sites, and improved interactions with the analyte.^16^ Among the diverse classes of nanomaterials explored for sensing applications, carbon-based nanomaterials offer critical advantages including high surface-to-volume ratios, tunable physicochemical properties and the possibility to amplify detection signals, improving both sensitivity and selectivity toward target analytes.^17,18^ In particular, carbon nanoparticles (CNPs) have emerged as versatile and sustainable platforms for environmental sensing,^19,20^ combining excellent electronic properties, low toxicity and facile surface functionalization.^21^ Their synthesis through green chemistry approaches makes them economically and environmentally advantageous compared to metal-based nanostructures,^22^ employing readily available precursors such as citric acid, carbohydrates or biomass waste.^23^ CNPs exhibit tunable fluorescence emission originating from surface states and core electronic transitions, which can be modulated through structural design, heteroatom doping, and surface chemistry modification.^24^ The abundance of oxygen-containing functional groups (carboxyl, hydroxyl, carbonyl) on the CNPs surface provides reactive sites for covalent grafting of molecular recognition units,^25^ enabling the rational design of chemically selective sensors for target analytes, including aldehydes, metal ions, and other environmental pollutants.^26,27^

Recent literature points out different fluorescence sensing strategies for FA detection,^12^ including molecular organic probes based on mechanisms such as aza-Cope rearrangement and Schiff base formation on the covalent side, polymeric systems addressing limitations of solubility in water,^28,29^ and metal–organic frameworks offering high potential as adsorbents for air monitoring.^30,31^ Despite these advantages, challenges remain including complex synthesis protocols, limited photostability of many molecular probes, and selectivity issues in the presence of structurally similar aldehydes and interferents. This underlines the need for sensing platforms that combine simple preparation, robust optical properties, good aqueous compatibility, and effective selectivity toward FA.

Here we present a fluorescent sensing platform based on carbon nanoparticles functionalized with diethanolamine (CNPs-DEA) for the detection of FA in aqueous medium. Comprehensive characterization was performed to establish the morphological, chemical and optical properties of the synthesized CNPs. The sensing capabilities of CNPs-DEA were systematically evaluated in aqueous solution, where quantitative detection performance and selectivity were assessed. Additionally, preliminary qualitative studies were conducted in solid state to explore the applicability of CNPs-DEA for gaseous FA detection, also in real samples demonstrating their potential for practical indoor air quality monitoring applications.

## 2 Results and Discussion

### 2.1 Design and Synthesis of CNPs-DEA

The choice to functionalize the CNPs with diethanolamine was born from the goal of creating a fluorescent nanosensor capable of selectively recognizing FA. In this design, the nanoparticle core fulfills two functions: it provides the intrinsic fluorescence thanks to its characteristic emission profile, and it serves as a versatile platform for multivalent functionalization. On the other side, the rationale for selecting diethanolamine (DEA) lies in the specific spatial orientation of the two terminal hydroxyl groups, which provide a recognition motif able to form two hydrogen bonds with the oxygen atom of FA. The flexible ethyl chains of DEA enable the two hydroxyl groups to cooperative bind FA, significantly increasing the affinity for it through a chelate-link effect. This strategy allows us to attach multiple recognition units on a single nanostructure, thereby significantly enhancing selectivity toward FA. Moreover, functionalization with diethanolamine groups is expected to increase the hydrophilic character of the carbon nanoparticles (when compared to the dopamine moiety used in our precedent work^27^), leading to enhanced aqueous solubility and stability over time. This behavior supports solution-based handling and storage, while reducing the tendency toward aggregation into larger clusters.

The final CNPs were achieved through three synthetic steps (Scheme 1). Initially, CNPs-COOH were produced by subjecting citric acid to solvent-free carbonization at 300 °C, generating a dark brown residue that was then dissolved in an aqueous NaOH solution. Purification was performed by centrifugation, to discard larger particulate matter, followed by dialysis to remove low-molecular-weight species, affording CNPs with uniform morphology. Because of the intrinsic structure of citric acid, the obtained CNPs present abundant surface carboxyl groups, which serve as reactive moieties for later modification (Scheme 1a). The second step involved the activation of these functionalities through the reaction with pentafluorophenol (PFPh), which acted both as reagent and solvent, in the presence of a carbodiimide as coupling agent (Scheme 1b). Being soluble in CH_2_Cl_2_, the resulting CNPs-PFPh enable nucleophilic substitution with the recognition site. The last stage involved the functionalization of CNPs-PFPh with diethanolamine, using DIPEA to enhance the nucleophilicity of the amine moiety (Scheme 1c). The reaction mixture underwent dialysis in water for 2 days, yielding purified CNPs-DEA.

**Scheme 1:**
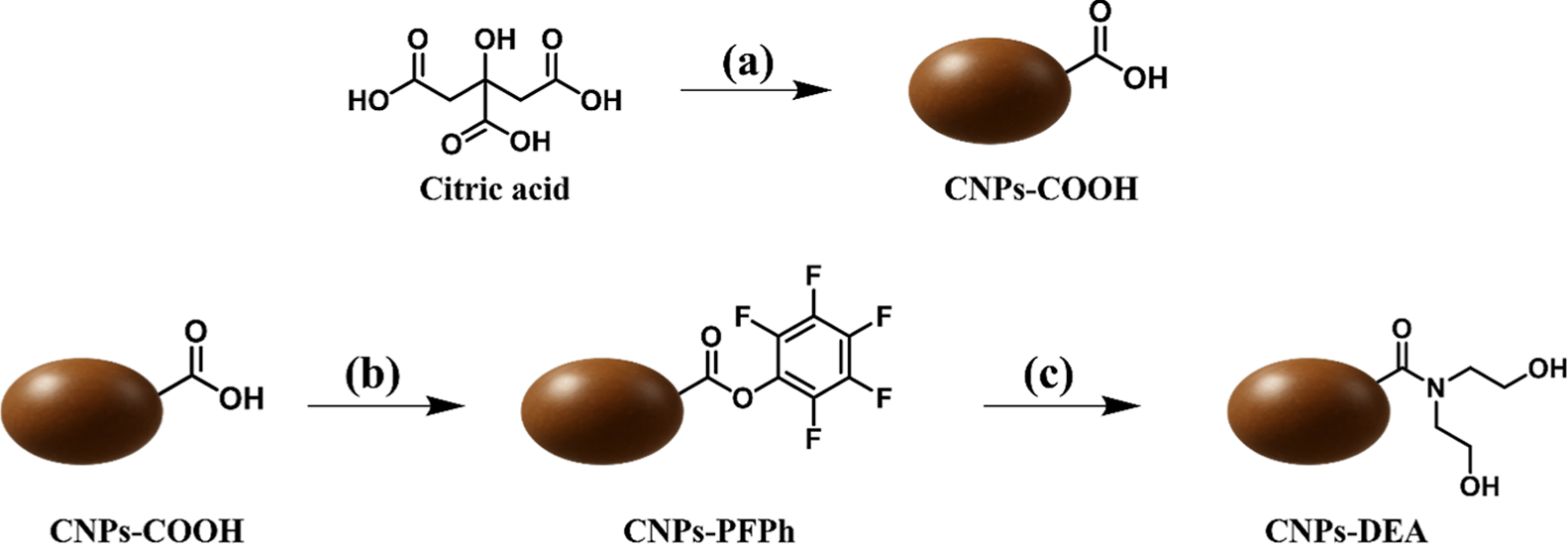
Schematic Pathway Showing the Reactions to Obtain CNPs-DEA: (a) 300 °C, NaOH_aq_ 0.1 M, 30 min; (b) Pentafluorophenol, EDC Hydrochloride, 50 °C, N_2_, 48 h; (c) Diethanolamine, DIPEA, CH_2_Cl_2_, Room Temperature, N_2_, 3 Days

### 2.2 Characterization of CNPs-DEA

Once synthesized, CNPs-DEA were characterized to investigate chemical structure, morphology and optical behavior.

#### 2.2.1 Chemical Characterization (XPS, ^1^H NMR and FT-IR)

Chemical characterization was extensively performed using spectroscopic techniques to verify the success of functionalization. X-ray photoelectron spectroscopy (XPS) was employed to investigate the electronic structure of the nanoparticles and confirm surface functionalization with diethanolamine. The XPS survey spectrum of CNPs-DEA is shown in Figure S1. The XP spectrum of CNPs-DEA in the C 1s region shows three main experimental peaks at 284.5, 285.7, and 288.1 eV (Figure 1a). Deconvolution of the spectrum required five Gaussian components at: 284.5 eV (C sp^2^ states, relative area 19.8%), 285.0 eV (C sp^3^ states, including C–C, C–H, and adventitious carbon, relative area 22.5%), 285.7 eV (C–N states, relative area 25.7%), 286.3 eV (C–OH groups, relative area 25.7%), and 287.8 eV (–C=O groups, relative area 6.3%).^32,33^ The intensity ratio for the C sp^2^/C sp^3^/C–N/C–OH/O=C–N contributions is 2.2/2.4/3/3/1. Figure 1b shows the O 1s XP spectrum of CNPs-DEA. A broad peak centered at 533.7 eV is evident. Spectrum fitting required three Gaussians at: 531.8 eV (O=C–N states, relative area 18.0%), 533.3 eV (C–OH groups, relative area 36.1%), and 533.8 eV (substrate oxygen, SiO_2_, relative area 45.9%).^34^ The intensity ratio of O=C–N to C–OH is approximately 1:2, consistent with the structure of CNPs-DEA. The N 1s XP spectrum is reported in Figure 1c, showing a broad band peak centered at 399.9 eV. A careful deconvolution resolved two components at: 399.8 eV (N–C=O amide states, relative area 76.6%) and 401.6 eV (quaternized nitrogen, likely because of amide protonation, relative area 23.4%).^35,36^ The relative intensity percentage of N–C=O and N^+^ species is 77/23, respectively. However, this does not influence the sensing mechanism and performance.

**Figure 1:**
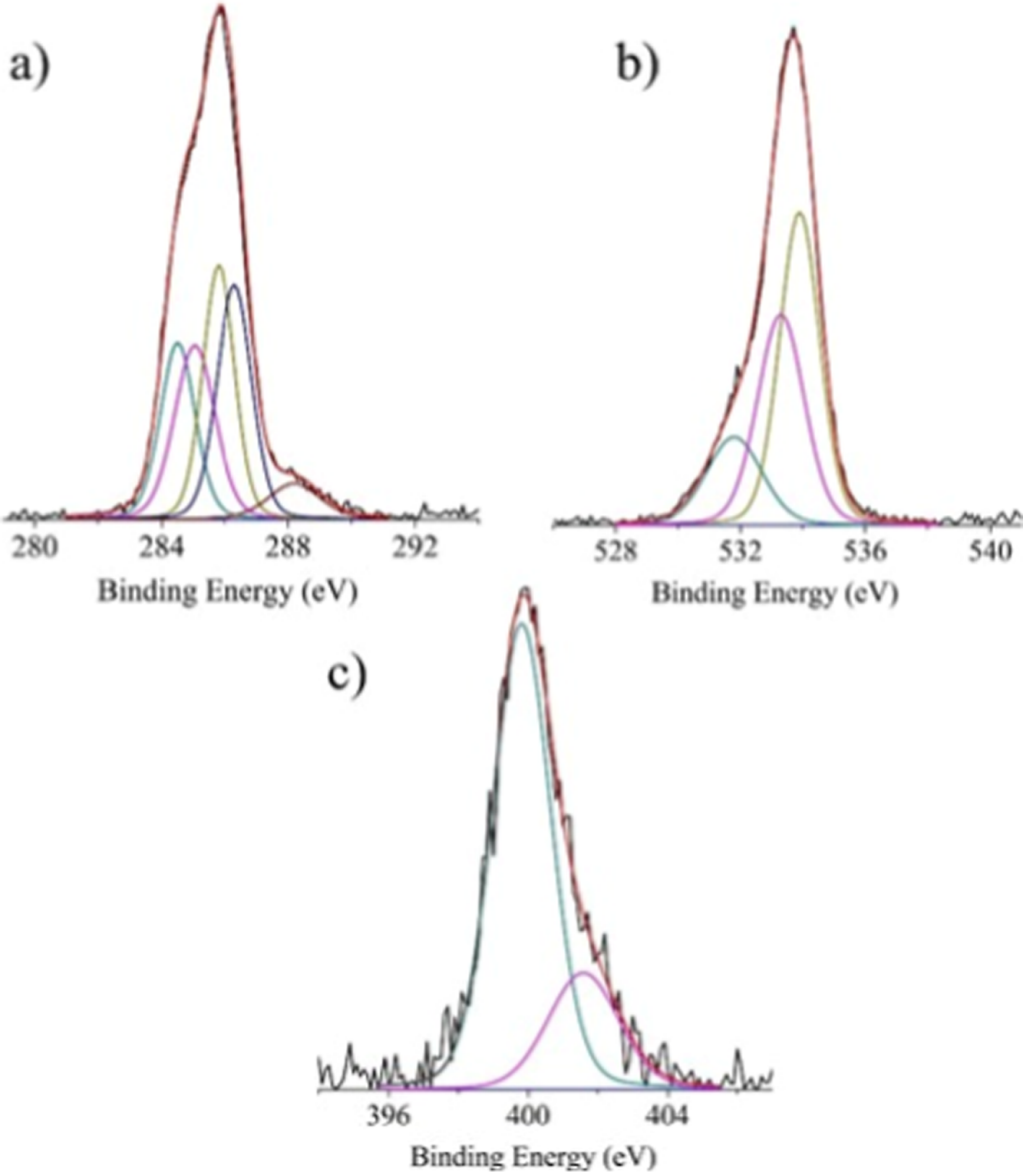
Al Kα excited XPS of the CNPs-DEA in (a) C 1s binding energy region, with Gaussian components at 284.5, 285.0, 285.7, 286.3, and 287.8 eV (dark cyan, magenta, dark yellow, navy, and winer respectively); (b) O 1s binding energy region, with components at 531.8, 533.3, and 533.8 eV (dark cyan, magenta, and dark yellow, respectively); (c) N 1s binding energy region, N 1s region, with components at 399.8 and 401.6 eV (dark cyan and magenta, respectively). In all spectra, the blue line represents the background, while the red line corresponds to the fitted envelope obtained from the sum of the Gaussian components, superimposed on the experimental data (black line).

The ^1^H NMR spectrum of CNPs-DEA, recorded in D_2_O, displays the protons relative to the diethanolamine moiety, whose signals moved to downfield with respect to the free diethanolamine in water (Supporting Information, Figure S2). This shift is consistent with amide bond formation and therefore indicative of effective nanoparticle functionalization with diethanolamine.

FT-IR measurements were carried out to verify successful functionalization. By comparing the spectra of CNPs-PFPh and CNPs-DEA (Supporting Information, Figure S3) the absence of the bands at 996 and 1009 cm^–1^, assigned to the C–F stretching of PFPh, clearly indicates that the substitution reaction has taken place. Further confirmation of amide formation is provided by the band at 1622 cm^–1^, which can be attributed to the C=O stretching vibration of a tertiary amide.

#### 2.2.2 Morphological Characterization (AFM and TEM)

Figure 2b,c show representative TEM images of CNPs-DEA, highlighting two distinct morphological populations. In Figure 2b, the nanoparticles appear predominantly as aggregated structures, whereas Figure 2c shows particles with a higher degree of dispersion, exhibiting a quasi-circular shape and sizes ranging from approximately 40–70 nm. Further structural characterization by HRTEM reveals that both the aggregated CNPs (Figure 2d) and the well-dispersed nanoparticles (Figure 2e) exhibit a crystalline nature. In the case of the aggregates, a polycrystalline structure is observed, with multiple crystalline domains oriented differently, which makes an accurate determination of the interplanar spacing challenging. By contrast, the dispersed CNPs display clearer lattice fringes (Figure 2g), allowing the measurement of a *d*-spacing of approximately 0.25 nm, consistent with in-plane graphitic ordering. The three concentric rings observed in the FFT pattern can be tentatively assigned to the (002), (100), and (110) reflections of turbostratic graphite (Figure 2h), confirming the polycrystalline nature of the CNPs and the presence of randomly oriented graphitic domains. The AFM imaging (Figure 2a) supports the TEM results, as isolated CNPS-DEA are characterized by a disk-like morphology with a height of 2 ± 1 nm, in agreement with previous findings.^22^ These results demonstrate that the DEA functionalization does not alter the particle morphology.

**Figure 2:**
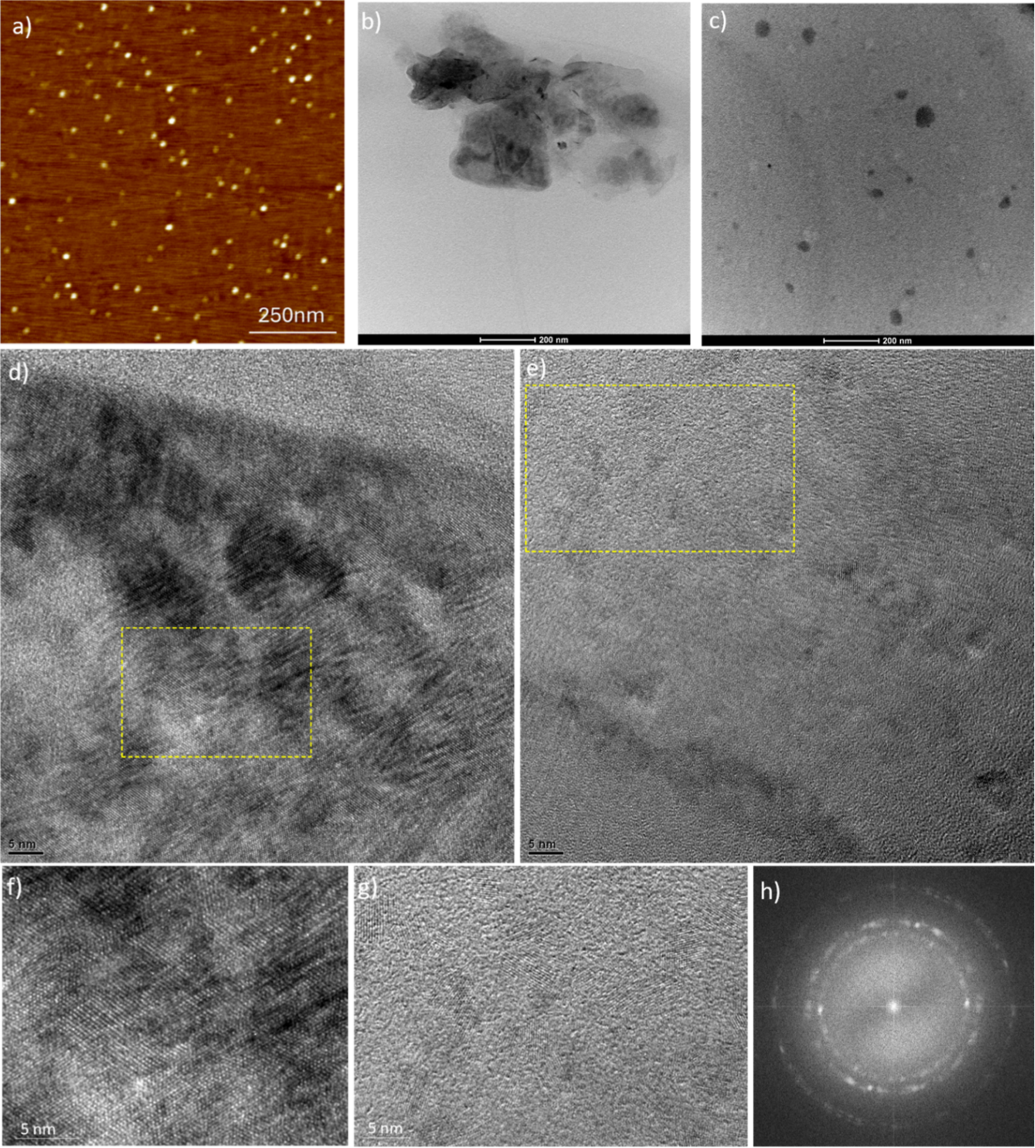
(a) The AFM image of CNPs-DEA reveals the expected disk-like morphology. (b,c) Representative TEM images of CNPs–DEA showing two distinct morphologies: (b) aggregated carbon nanoparticles and (c) well-dispersed particles with a quasi-spherical geometry. (d,e) High-magnification HRTEM images: (f) High-magnification HRTEM image of the region marked in (d), highlighting the polycrystalline structure of the aggregated nanoparticles. (g) High-magnification HRTEM image of the region marked in (e), showing well-defined lattice fringes in the dispersed CNPs. (h) Corresponding FFT pattern confirming the polycrystalline character of the sample and the presence of randomly oriented graphitic domains.

#### 2.2.3 Optical Characterization (UV–Visible and Fluorescence Spectroscopy)

The optical behavior of CNPs-DEA was evaluated through UV–vis and fluorescence spectroscopy. The absorption spectrum was recorded using a concentration of 0.1 mg mL^–1^ in Milli-Q water; it reveals a broad absorption feature arising from the CNPs, which can be attributed to π–π* and *n*-π* electronic transitions of conjugated C=C domains (Figure 3a). This wide absorption profile is typical of graphitic carbon nanoparticles, whose weak interlayer interactions and propensity to adopt low-energy conformations generate substantial structural disorder, ultimately resulting in band broadening. Fluorescence spectra of CNPs-DEA were recorded using a concentration of 0.1 mg mL^–1^ in Milli-Q water (Figure 3b), over the λ_ex_ range 300–400 nm. The emission maximum is centered at 455 nm and remains unchanged independently from λ_ex_, showing a quantum yield of 0.13%. This behavior can be related to a reduced polydispersity, due to the dialysis process, also confirmed by TEM and AFM analyses, thus also reducing the emissive states. This, in addition to the significant dimensions of these CNPs, may have contributed to an excitation-independent emission. Besides the main emission feature, two additional wavelength regions provide further insight into the system’s behavior. One is a narrow band that systematically follows the excitation wavelength, which is characteristic of water Raman scattering and is routinely observed in fluorescence experiments. The other is a weak shoulder centered around 500 nm that remains unchanged regardless of λ_ex_, that originates from the functional groups decorating the nanoparticle surface.

**Figure 3:**
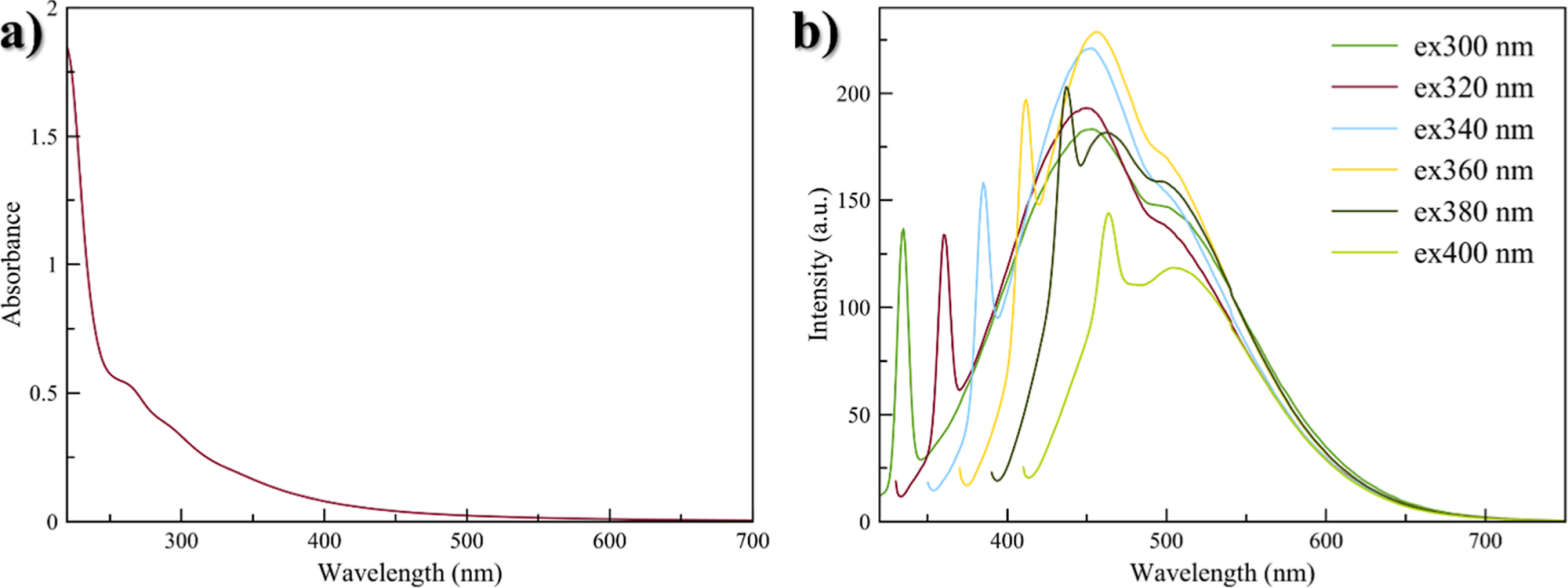
Optical characterization of CNPs-DEA: (a) UV–visible spectrum (0.1 mg mL^–1^ in Milli-Q water) and (b) fluorescence spectra in the excitation range 300–400 nm (0.1 mg mL^–1^ in Milli-Q water).

### 2.3 FA Sensing in Solution

#### 2.3.1 Fluorescence Titration with FA

The sensing performance of the synthesized nanosensor was initially examined in water (pH = 7). Fluorescence titration was carried out in water at pH 7, by adding increasing aliquots (from 2 to 100 μL) of a 10^–4^ M FA solution in water to a cuvette containing CNPs-DEA 0.5 mg mL^–1^. After dilution in the cuvette, these additions correspond to an FA concentration range of approximately 0.1–5 ppm. Upon incremental addition of FA, the CNPs-DEA solution exhibited a progressive decrease in fluorescence intensity (Figure 4a), corresponding to a 12.1% reduction in quantum yield, after addition of 5 ppm of FA. This behavior allowed us to define a linear range between 0.1 and 1.4 ppm of FA, obtaining a calibration curve (Figure S4). It shows that the standard deviation for each concentration is significantly lower than the signal change induced by FA, ensuring that the detected variations are meaningful and not due to experimental fluctuations. The strength of the interaction between CNPs-DEA and FA was quantified by determining the apparent binding constant, giving a log β value of 4.31 ± 0.01. To evaluate the sensitivity of the probe, the expression LOD = 3σ/k was used, assuming as σ the standard deviation on 10 different measurements of the blank, and k as the slope of the calibration curve in the linear range (Figure S4). Following this approach, the detection limit was determined to be 46 ppb. This sensitivity is considerably higher than required for practical applications, as it falls nearly an order of magnitude below the World Health Organization guideline of 900 ppb for FA in drinking water. Such performance highlights CNPs-DEA’s potential for reliable FA monitoring in aqueous environments.

**Figure 4:**
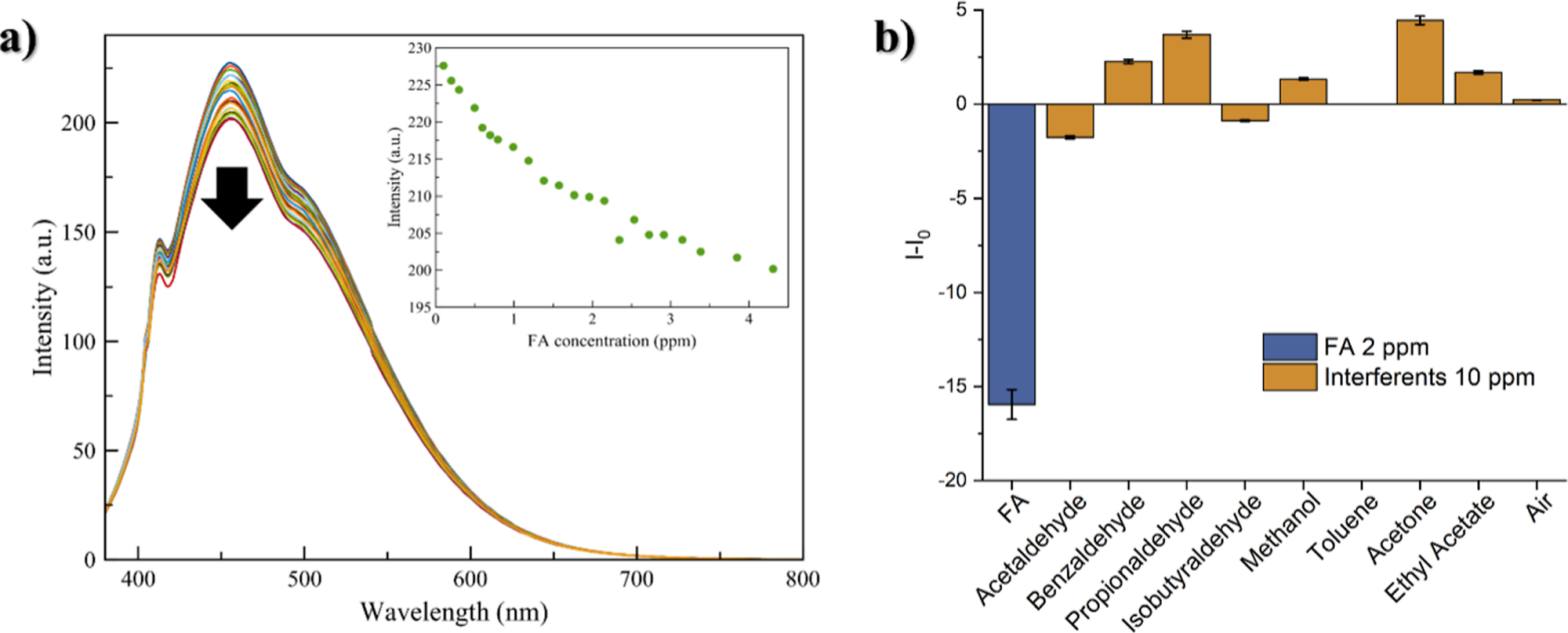
(a) Emission spectra of CNPs-DEA (0.5 mg mL^–1^ in Milli-Q water, λ_ex_ = 360 nm, λ_em_ = 456 nm) upon progressive addition of FA, in concentration range 0.1–5 ppm. The inset represents the fluorescence intensity of CNPs after gradual addition of FA. (b) Histogram with selectivity tests results: response of CNPs-DEA to FA (blue) and excess of interferents (orange).

#### 2.3.2 Selectivity

Assessing selectivity is a crucial step to verify that the sensor can distinguish the target analyte from other species commonly encountered in real samples. For this purpose, a series of interference experiments were conducted in water by exposing CNPs-DEA to large excesses (10 ppm) of various potentially competing compounds. Two main classes of interferents were used: small aldehydes structurally related to FA (namely acetaldehyde, propionaldehyde, *iso*-butyraldehyde, and benzaldehyde) and representative VOCs, such as methanol, acetone, ethyl acetate, and toluene. In addition, atmospheric air was evaluated as interferent by gently bubbling it through the sample; the gas mixture contains significant amounts of water vapor (∼24,000 ppm) and trace gases such as CO_2_ (400 ppm), NO (5 ppm), and CO (10 ppm). As illustrated in the histogram in Figure 4b, none of these species induced an emission variation comparable to that produced by 2 ppm of FA, demonstrating the high selectivity of CNPs-DEA toward the target analyte. The procedure for the preparation of the interferents is reported in Supporting Information, as well as the comparison of full emission spectra per interferent (Figure S6a–i). To further evaluate the robustness of the CNPs-DEA sensor, competitive selectivity experiments were performed. The fluorescence response was recorded for the sensor exposed to each individual interferent (10 ppm) and, subsequently, to a mixture containing both the interferent (10 ppm) and FA (2 ppm). As shown in Figure S7, the potentially completing VOCs did not significantly affect the characteristic quenching induced by FA, confirming that the probe maintains its high selectivity even in more complex chemical environments.

#### 2.3.3 Sensing Mechanism

The rationale behind the sensing mechanism was elucidated by performing a DFT study. As reported in our previous work,^27^ two graphene surfaces (1.2 × 1.1 nm), positioned 0.3 nm apart, were studied to mimic the CNPs core (Figure S8a). The addition of two diethanolamine units at the borders of the CNPs provides a model system for the receptive material (CNPs-DEA). A conformational screening on CNPs-DEA reveals different relative orientations of the diethanolamine units, characterized by intramolecular hydrogen bonds (HBs). Two main conformations were identified (Figure S8b,c—Conformer A and B, respectively), in which the carbonyl groups of the amide moieties are either facing each other (Conformation A) or arranged parallel to each other (Conformation B). Surprisingly, conformation A is approximately 30.1 kcal/mol more stable than conformer B, owing to reduced steric hindrance and the formation of an intramolecular HB between the aminoethanol functional groups (Figure S8a).^37,38^ The optimized CNPs-DEA system was used to investigate the sensing mechanism toward formaldehyde (FA). Owing to its electronic structure, two different electron-density regions can be found in FA: (i) a partially negatively charged region localized on the carbonyl oxygen (C=O), and (ii) a partially positively charged region on the aldehyde hydrogen (O=C–H) (Figure S8d). In light of this, FA can be considered both a good hydrogen bond donor and acceptor, representing a perfect match for the CNPs-DEA system due to the numerous hydroxyl groups present in its structure. Indeed, three hydrogen bonds are found in the complex between CNPs-DEA and FA (Figure 5). Specifically, two hydrogen bonds are formed directly between the receptor and FA: one between a hydroxyl group of the receptor and the carbonyl group of FA (C=O---HO), and one CH hydrogen bond between the electropositive aldehyde hydrogen and the hydroxyl group (HO---HCone between a hydroxyl groupO) (Figure 5). The hydrogen-bonding network established upon complexation, consisting of three HBs, ensures strong affinity between FA and the receptor. This is further supported by the complexation energy (*E*_complex_) of 10.7 kcal/mol for the FA-CNPs-DEA complex.

**Figure 5:**
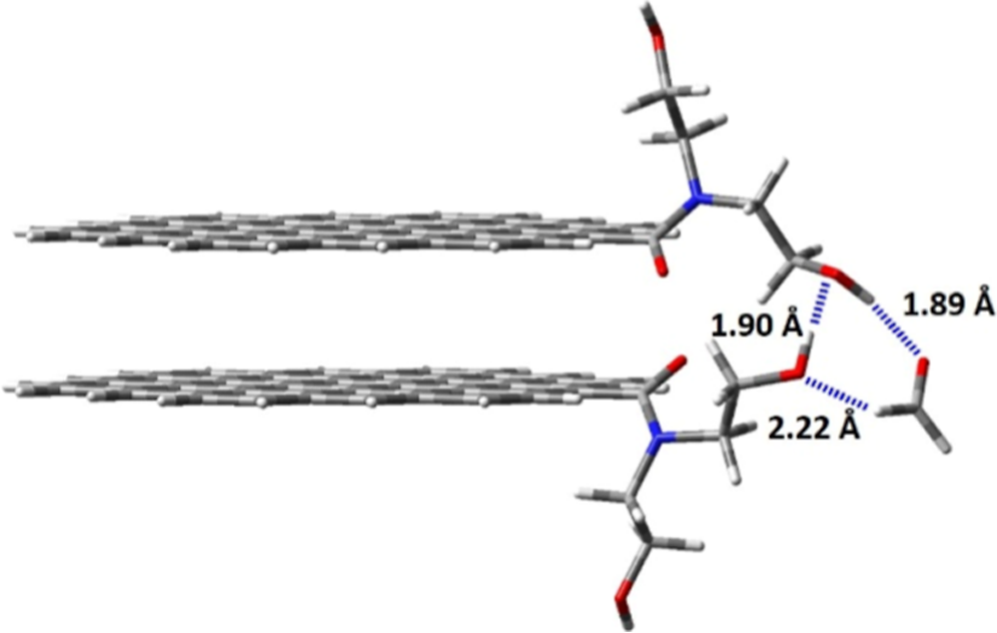
Optimized structure of the complex between CNPs-DEA and formaldehyde. Dashed blue lines represent noncovalent interactions, mainly hydrogen bonds (HBs), which length in reported in angstrom (Å).

The reported *E*_complex_ value is in agreement with the experimentally measured log β value of 4.31 ± 0.01 for FA-CNPs-DEA, as supported by similar studies reported in the literature.^27^ The strong affinity toward FA is further highlighted by the *E*_complex_ value of 5.9 kcal/mol calculated for a water dimer (2H_2_O)—approximately 4.8 kcal/mol lower than the value found for the FA-CNPs-DEA complex (Supporting Information, Table S3).

At the current stage, the exact photophysical pathway responsible for the fluorescence modulation cannot be unambiguously assigned. The experimental evidence and DFT calculations support a supramolecular interaction between formaldehyde and the diethanolamine-functionalized nanoparticles through hydrogen bonding. This interaction alters the local environment of the emissive carbon nanoparticles, resulting in the observed fluorescence variation. A deeper investigation of the excited-state processes would require time-resolved spectroscopic studies, which are beyond the scope of the present work.

### 2.4 Gaseous FA Detection by Strip Test

Gas-phase detection experiments were designed based on the liquid–vapor equilibrium of aqueous FA solutions,^39^ using a customized setup to expose the sensor to controlled vapor concentrations. A polyamide membrane served as the support, having the size to match the inner diameter of a 20 mL vial cap and coated with CNPs-DEA via drop-casting (1 μL, 1 mg mL^–1^ in Milli-Q water). Once drop casted onto the solid support, CNPs-DEA show high stability over 15 days (Figure S10a). In this configuration, sealing the vial positions the sensing strip directly in contact with the headspace, consisting of ambient air. This environment inherently contains a mixture of gases, including CO_2_ (400 ppm), NO (5 ppm) and CO (10 ppm). Fluorescence FA detection was performed using a commercial smartphone coupled with a 3D-printed dark chamber and a UV LED (365 nm excitation), with images acquired before and after exposure to gaseous FA (Figure 6a). Images were processed using ImageJ software to extract the average fluorescence intensity in the RGB channel. This platform was utilized to investigate both the response kinetics and the working range of the sensor toward gaseous FA. All experiments performed on solid supports were carried out in triplicate using independently prepared membranes, and the reported values correspond to the average of these independent measurements. The good reproducibility observed among the different experiments indicates that the fluorescence response is not influenced by localized regions with higher probe concentration, confirming the macroscopic uniformity of the CNP-loaded polyamide substrates. All theoretical considerations and fabrication protocols are detailed in the Supporting Information.

**Figure 6:**
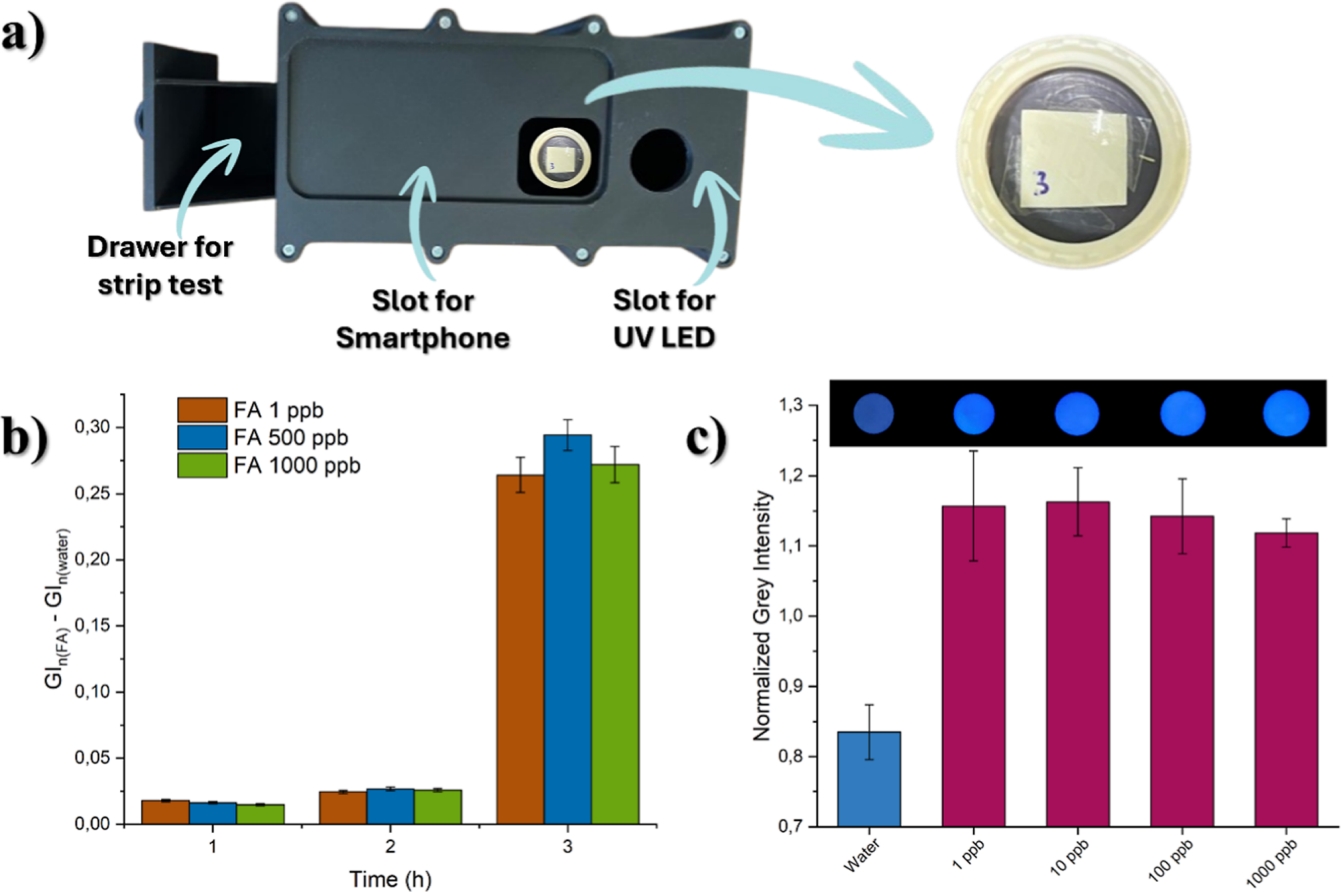
(a) Real image of the 3D-printed dark chamber, including proper slots for the UV LED, the smartphone and a drawer to place the sensor at a fixed position from the camera. The zoom represents an image of the solid-state sensor placed in the inner surface of a vial’s cap. (b) Histogram of the normalized gray intensity of the CNPs-DEA sensor, expressed as the difference between the response to formaldehyde (GI_*n*(FA)_) and the response to water (GI_*n*(water)_), measured over 3 h of analysis, for three different gaseous FA concentrations (1 ppb, 500 and 1000 ppb). Error bars refer to the standard deviation on the replicates. (c) Histogram representing the values obtained from image analysis of CNPs-DEA strip test after 3 h exposure to water (blue) and different concentrations of gaseous FA (purple). Error bars represent the standard deviation over the replicates. Real images of the sensor under UV LED are displayed above each bar, showing the visual change from the blank (water) to increasing FA concentrations.

#### 2.4.1 Kinetics Studies

Kinetics studies were performed to define the response time of the strip test to the FA vapors. To this end, 12 vials were prepared to allow for independent measurements at three different time intervals (1 h, 2 h and 3 h). Specifically, three vials three contained Milli-Q water as control (one for each time point), while the remaining nine vials contained aqueous solutions adjusted to generate gaseous FA concentrations of 1 ppb, 500 and 1000 ppb in the headspace, three replicates for each concentration, each corresponding to a specific exposure time. A sensing strip was positioned in each vial, with images acquired prior to sealing (*t* = 0). To monitor the signal evolution, the 12 vials were processed in sets corresponding to each time point. Specifically, for each interval, one control vial and three vials containing different concentrations (1 ppb, 500 ppb and 1 ppm) were unsealed, and the strips were photographed immediately upon retrieval. The study revealed that, after 1 h of exposure, no appreciable change in fluorescence intensity was observed, for any of the tested conditions. From the second hour onward, the contribution of water remained constant, confirming that the saturated water vapor atmosphere within the vials does not interfere with the sensing response, while a distinct signal attributable to FA emerged after 3 h of exposure, for all the tested concentrations (Figure 6b). This time frame is thought to be largely influenced by the experimental conditions, particularly the time required for FA to partition from the aqueous solution into the headspace of the vial. The 3 h interval was therefore selected to ensure that the response reflects a stabilized gaseous FA concentration.

#### 2.4.2 Qualitative FA Detection

Following kinetic optimization, the detection range was evaluated by exposing the CNPs-DEA solid sensors to nine distinct FA vapor concentrations: 1, 2, 5, 10, 50, 75, 100, 500 ppb, and 1 ppm, respectively. The lowest concentration (1 ppb) was selected based on the linearity range reported in the reference study,^39^ below which reliable quantitative evaluation was not possible due to the analytical setup. The strips were placed in sealed vials maintained at a controlled temperature of 25 °C and, based on the kinetic results, images were taken after 3 h of exposure (experiments performed at +4 °C showed no significant sensing performance).

Remarkably, image analysis revealed that the CNPs-DEA sensor could clearly distinguish the presence of FA, also in the presence of a large excess of water (the first bar shows the response to water control) even at the lowest tested concentration of 1 ppb (Figure 6c). Probably, the lower LOD obtained in gas phase can be ascribed to the absence of the solvent respect to the LOD value (46 ppb) calculated in water. However, the fluorescence response did not exhibit a linear dependence on analyte concentration, suggesting a threshold mechanism under these conditions. Consequently, while CNPs-DEA does not provide quantitative detection in this range, it functions as an exceptional qualitative probe. Also, the gases present in ambient air (and in the headspace of the vial) did not interfere with the fluorescence response of CNPs-DEA. This confirms that the recognition mechanism is highly specific for FA even in the presence of coexisting atmospheric trace gases. These results highlight a significant advantage of the developed strip test: the ability to detect trace levels of FA (down to 1 ppb) in saturated humidity environments, effectively discriminating the analyte from common air interferences. Crucially, such high-performance detection is accessible using a commercial smartphone.

A comparative analysis with state-of-the-art literature highlights the competitive positioning of the CNPs-DEA sensor (Table 1).^12^ Covalent molecular organic probes ensure high specificity through targeted chemical reactions, yet they are often affected by complex multistep synthesis, poor water solubility and limited photostability.^40–42^ Conversely, more advanced architectures, such as metal–organic frameworks (MOFs), leverage superior surface area and porosity for analyte preconcentration, achieving low detection limits; however, their practical deployment is often hindered by high fabrication costs and use of transition metal atoms (e.g., use of lanthanides), intricate synthesis, and the need for laboratory readout equipment.^43–45^ Similarly, recent polymeric sensors obtained by De et al.^46,47^ can achieve rapid response kinetics and competitive limits of detection, competing with solution-phase probes while offering superior mechanical stability for portable devices. Notably, a recent study reported carbon nanoparticles functionalized with dopamine, exhibiting a linear response to gaseous FA in the concentration range between 10 ppb and 1 ppm.^27^ In particular, if compared with the latter example, the nanosensor here reported shows a LOD lower by 1 order of magnitude (1 ppb), leading to better sensitivity. Furthermore, as anticipated, the introduction of diethanolamine moieties significantly improves the aqueous solubility of the CNPs, enabling more reliable handling in solution, facilitating titration experiments, and allowing stable storage while minimizing the formation of large aggregates. An additional advantage lies in the practical usability of the system: although CNPs-DEA do not display a strictly linear response as a function of FA concentration, a clear turn-on signal is observable even with FA traces by the naked eye under UV LED irradiation.

**Table 1:** Literature Comparison of Fluorescent Sensors for FA Detection

sensing element	LOD	fluorescence readout	source	refs
molecular probe	0.3 ppb	fluorimeter	air, seafood	^40^
molecular probe	0.17 ppm	naked eye	solution	^41^
molecular probe	1.9 ppb	smartphone	air	^42^
MOF + CDs + AuNPs	0.013 ppm	smartphone	air, solution	^43^
MOF	5.83 ppb	naked eye	solution	^44^
MOF	18.6 ppb	naked eye	air, solution	^45^
polymeric probe	0.04 ppb	naked eye	air, solution	^46^
polymeric probe	51 ppb	fluorimeter	solution	^47^
functionalized nanoparticle	10 ppb	smartphone	air, solution	^27^
functionalized nanoparticle	1 ppb	smartphone	air, solution	this work

Within this context, the CNPs-DEA sensor offers distinct features, through its operational simplicity and cost-effectiveness. By combining a simple surface functionalization with a common readout device, CNPs-DEA provides a valid alternative to address the critical gap between sophisticated molecular design and the practical need for accessible, field-deployable monitoring tools.

### 2.5 FA Detection in a Real Sample

The applicability of the CNPs-DEA sensor in real-world scenarios was tested on a commercial paint formulation. Given the complexity of the sample matrix, the standard addition method was employed on the pretreated solution to ensure accurate determination. Linear regression analysis of the fluorescence response versus spiked FA concentrations allowed for the precise quantification of the analyte (Supporting Information, Figure S11). Based on the data obtained from the diluted measurement solution (30 μM), the initial FA concentration in the paint was calculated to be 9 ppm. This demonstrates the sensor’s capability to operate effectively even in complex commercial mixtures.

## 3 Conclusion

In this work, we developed diethanolamine functionalized carbon nanoparticles (CNPs-DEA), with a simple synthetic protocol and low-cost precursors. The study provided crucial insights into the recognition mechanism. Supported by DFT calculations, our findings revealed that the nanoparticle surface acts as a nanoscale scaffold, creating a local proximity effect among the diethanolamine functional groups. This specific spatial arrangement enables the simultaneous recognition of a formaldehyde molecule by two adjacent sites, significantly enhancing the binding efficiency. Leveraging this cooperative interaction, the sensor exhibited remarkable performance in the liquid phase, achieving a LOD of 46 ppb in water. This sensing capability was successfully translated into a solid-state platform by drop-casting the CNPs on polyamide membranes. The resulting strip test demonstrated an ultralow detection threshold (1 ppb) for gaseous formaldehyde, combining high selectivity and humidity tolerance with the operational simplicity of a smartphone readout. Although the resulting response is qualitative, the combination of high sensitivity, low cost, and smartphone compatibility makes CNPs-DEA a tool for real-world applications compared to more complex, laboratory-confined alternatives.

## 4 Methods

### 4.1 Synthesis and Functionalization

Citric acid, diethanolamine, pentafluorophenol, EDC hydrochloride, NaOH and DIPEA were acquired by Sigma-Aldrich and used as received. Functionalization of the CNPs was carried out under nitrogen atmosphere to prevent the presence of moisture and the subsequent hydrolysis of the product. NMR measurement was performed at 27 °C on a Varian UNITY Inova 500 MHz spectrometer equipped with a pulsed-field gradient module (*Z* axis) and a tunable 5 mm Varian inverse detection probe (ID-PFG). Chemical shifts in ppm were referenced to the residual solvent signal, and the coupling constants are reported in Hz.

#### 4.1.1 Synthesis of CNPs-COOH

Powder citric acid (20 g, 0.1 mol) was placed in a beaker and heated to 300 °C on a hot plate at 300 °C until caramelization took place. After cooling down to room temperature, 100 mL of a 0.25 M NaOH aqueous solution was slowly added while stirring. The resulting mixture was centrifuged at 3000 rpm for 30 min and the supernatant was transferred into a dialysis membrane (cutoff 12–14 kDa). After 2 days of dialysis, the retained solution was dried under reduced pressure, yielding CNPs-COOH.

#### 4.1.2 Synthesis of CNPs-PFPh

CNPs-COOH (275 mg) were dissolved in 5 mL of pentafluorophenol (PFPh) at 50 °C under stirring. After complete solubilization, EDC × HCl (880 mg, 5 mmol) was added under nitrogen atmosphere. After 48 h, the mixture was allowed to cool to room temperature and diluted with 25 mL of dichloromethane. A liquid–liquid extraction was then performed, using water at pH around 9. The organic phase was dried under vacuum, obtaining CNPs-PFPh, which were characterized by FT-IR spectroscopy.

#### 4.1.3 Synthesis of CNPs-DEA

CNPs-PFPh (830 mg) were dissolved with 5 mL of dry CH_2_Cl_2_. Then, 379 μL of diethanolamine (4 mmol) and 3.44 mL of DIPEA (20 mmol) were added to the solution, and the reaction mixture was stirred at room temperature under nitrogen for 3 days. The solvent was removed under vacuum, and the solid was solubilized in water. The solution was transferred into a membrane (cutoff 12–14 kDa), to perform 3 days dialysis for further purification of the nanoparticles. The solution inside the membrane was then collected, and water evaporated under vacuum. Characterization of CNPs-DEA was performed using AFM, TEM, ^1^H NMR, XPS, FT-IR, UV–vis and fluorescence. ^1^H NMR (500 MHz, D_2_O, δ): 3.70 (t, *J* = 5.3 Hz, 4H; CH_2_–N), 3.06 (t, *J* = 5.3 Hz, 4H; CH_2_–O) ppm.

### 4.2 XPS Characterization

X-ray photoelectron spectra (XPS) were performed using a PHI 5000 Versa Probe II spectrometer (ULVAC-PHI, Inc.), operating under ultrahigh vacuum conditions (P ≈ 1 × 10^–8^ Pa), and using a monochromated Al Kα radiation.^32,48^ Spectra were collected at a takeoff angle of 45°, with respect to the Si substrate surfaces on which the samples were deposited. The pass energy was set to 5.85 eV, thus obtaining an instrumental energy resolution ≤0.5 eV. The acquired spectra were processed after subtraction of the background using the Shirley method.^32,48^ The binding energy scale was calibrated by fixing the Ag 3d_5/2_ peak of a clean silver reference at 368.3 eV.^49^ Quantitative atomic concentrations were determined by applying the corresponding atomic sensitivity factors. Detailed spectral deconvolution was carried out for selected core levels using the XPSPEAK 4.1 software. After background correction, the experimental spectra were fitted using Gaussian envelopes. The fitting procedure was based on a least-squares minimization approach to obtaining the best match between calculated and experimental profiles. The goodness of fit was evaluated through the residual factor *R*, defined as *R* = [Σ (*F*_obs_ – *F*_calc_)^2^/Σ (*F*_obs_)­2]^1/2^, which converged to a value of 0.03. The relative contribution of each chemical state was calculated from the integrated areas of the fitted components.

### 4.3 FT-IR Measurements

FT-IR measurements were performed using a PerkinElmer spectrum one spectrophotometer for CNPs-PFPh and CNPs-DEA. The samples were dispersed in KBr and pressed into pellets (sample/KBr ratio of 1:100). Transmission spectra were collected over the range 4000–450 cm^–1^, averaging 16 scans for each sample.

### 4.4 Morphological Characterization (AFM and TEM)

Morphological analysis was performed in tapping mode using a Nanoscope IIA-MultiMode atomic force microscope (AFM) from digital instruments, Santa Barbara, CA, USA. Images were recorded at a scan rate of 1 Hz, with a resolution of 512 × 512 pixels, employing Tap 300 G silicon probes (Budget sensors) mounted on cantilevers with a nominal force constant of 40 N m^1–^ and a resonant frequency of 300 kHz. AFM samples were prepared by rapidly drop-casting a few microliters of a previously diluted (0.1 mg/mL) aqueous dispersion of CNPs-DEA, onto freshly cleaved mica substrates.

TEM characterization was performed on a Tecnai T20 microscope (200 kV) and a 300 kV FEG-TEM equipped with a SuperTwin lens (point resolution 1.9 Å, lattice resolution 0.19 nm). Samples were prepared by depositing 20 μL of suspension onto carbon-coated copper grids (200 mesh) and drying at room temperature for 4 h.

### 4.5 UV–Visible and Fluorescence Measurements

The UV–vis absorption profile of CNPs-DEA was measured with a JASCO V-750 UV–vis double-beam spectrophotometer (0.1 resolution), using quartz cuvettes with a 1 cm optical path. Analyses were performed in Milli-Q water at a concentration of CNPs-DEA equal to 0.1 mg mL^–1^, within the 220–700 nm spectral window.

Photoluminescence experiments were conducted at room temperature on a JASCO FP-8550 spectrofluorometer (resolution 0.5 nm). The emission was detected at a 90° angle relative to the excitation beam, using slit widths of 2.5 nm for both excitation and emission. Working concentration of CNPs-DEA employed for fluorescence measurements was 0.5 mg mL^–1^. For the emission screening, the λ_ex_ was scanned from 300 to 400 nm. Fluorescence titrations with FA were performed at a fixed excitation wavelength of 360 nm, using CNPs-DEA 0.5 mg mL^–1^, and incremental amounts (2–100 μL) of a 10^–4^ M FA solution in Milli-Q water were introduced directly into the cuvette. The apparent binding constant (log β) and the corresponding standard deviation were estimated using the HypSpec software (version 1.1.33),^50^ which processes spectrophotometric data by fitting them to a selected complexation model through least-squares minimization. This procedure allows determination of both the spectral contributions of individual species and their stability constants. The reliability of the fitting was verified by means of a χ^2^ test, which evaluates the statistical distribution of residuals. Satisfactory agreement is generally associated with χ^2^ values around or below 12;^51^ in all cases, values below 10 were obtained from three independent data sets. For these calculations, a CNPs-DEA concentration of 10^–8^ M was used, based on estimates described in the Supporting Information. Quantum yields were evaluated using quinine hemisulfate as the reference standard.^52^ Measurements were performed both for pristine CNPs-DEA and after exposure to 5 ppm of FA.

Control experiments were carried out to exclude possible artifacts that could influence the fluorescence response of the sensing platform. First, the potential contribution of inner-filter effects was evaluated by recording the UV–vis absorption spectra of CNPs-DEA in the absence and in the presence of FA within the investigated concentration range. No significant overlap between the absorption bands and the excitation/emission wavelengths was observed, indicating that the fluorescence modulation cannot be attributed to inner-filter effects. The influence of pH variations was also examined by performing fluorescence measurements over a pH range of 5–9 under otherwise identical conditions. The fluorescence intensity of CNPs-DEA remained essentially unchanged in the absence of FA, confirming that the sensing response is not affected by moderate pH fluctuations. To assess the possible role of ionic strength, experiments were conducted in the presence of increasing concentrations of NaCl (0–100 mM). No significant changes in the fluorescence signal were detected, demonstrating that the sensing response is not influenced by variations in ionic strength. Finally, photobleaching effects were investigated by continuously illuminating the sensing substrate under the same excitation conditions used during the measurements. The fluorescence intensity remained stable over the acquisition time, indicating that the observed fluorescence variation is not related to photodegradation of CNPs-DEA.

### 4.6 Computational Analysis of the Sensing Mechanism

Gaussian09 program package was selected to perform ab initio and density functional theory (DFT) calculations.^53^ Geometry optimizations of the reported systems were carried out at the B3LYP/6-31G­(d,p) level of theory. A conformational study was performed on all structures to ensure that the absolute minimum was identified. Frequency calculations were conducted to confirm the absence of imaginary (negative) frequencies. GaussView software was used as a graphic interface to represent the optimized structures. Zero-point energy (ZPE) was included in all results.

### 4.7 Gaseous FA Detection by Test Strips

Polyamide membranes (0.2 μm pore size) were cut into 1.2 cm^2^ squares and treated with UV/O_3_ to improve wettability. A CNPs-DEA solution (1 μL, 0.5 mg mL^–1^) was drop-casted onto the membranes and annealed at 80 °C for 1 h to ensure solvent removal and particle immobilization.

Measurements were performed in 20 mL sealed vials at 25 °C. Controlled FA vapor concentrations were generated from aqueous solutions based on the liquid–vapor equilibrium model reported by Dong and Dasgupta (see Supporting Information for equations and calculations).^39^ Fluorescence readout was carried out using a smartphone coupled to a custom 3D-printed dark chamber (λ_ex_ = 365 nm). Validation across different devices was carried out using three smartphones (Samsung A22, iPhone 13 and iPhone 13 Pro), all operating with the ProCam application. In combination with a dark chamber, this setup minimizes ambient light interference and prevents automatic exposure adjustments. To account for strip-to-strip variability and ensure statistical reliability, each strip was prepared with three separate CNPs-DEA spots, providing an internal triplicate for every single experiment. Furthermore, each measurement was independently repeated three times using different strips obtained by different batches of CNPs-DEA. This approach yielded a total of nine replicates for each data point, which were used to determine the mean values and the relative standard deviations. Images were processed using ImageJ software to extract the average fluorescence intensity in the RGB channel, as detailed in the Supporting Information. We define “detectable” a signal exceeding the background noise of the blank by 3σ, ensuring a 99.7% confidence level that the observed variation is due to the presence of FA.

To evaluate the reliability of the sensing platform, repeated measurements were performed under identical experimental conditions using independently prepared sensing substrates. The results showed consistent fluorescence responses with no observable false-positive or false-negative signals within the tested concentration range, confirming the robustness and reproducibility of the sensing system.

### 4.8 FA Detection in a Real Sample

Formaldehyde was extracted from a commercial paint sample (2 g) using ethanol (20 mL) via a sonication-assisted procedure (20 min) followed by 24 h of stirring, in accordance with literature protocols.^43^ Upon filtration through a 0.45 μm membrane, the clear filtrate was analyzed using the CNPs-DEA probe in solution. To rigorously account for nonspecific matrix quenching or enhancement effects, the standard addition technique was employed. The sample was spiked with increasing concentrations of FA (using 2, 10, and 20 μL of a 10^–2^ M standard solution), and the initial analyte concentration was determined via linear regression analysis of the fluorescence response.

## Supplementary Material



## Notes

The Supporting Information includes structural characterization (XPS, 1H NMR, FT-IR), calculation of CNPs-DEA molar concentration, HypSpec plot, selectivity tests, computational analysis of the sensing mechanism, details of gaseous FA detection by test strip, and analysis of real sample (PDF)
