# Supplementary material for: A Smartphone-Assisted Approach to Formaldehyde Detection Using Diethanolamine-Grafted Carbon Nanoparticles
Source: ACS Omega. 2026 Jun 5;11(24):35421–33. doi: 10.1021/acsomega.6c00964 (PMC13295052; doi:10.1021/acsomega.6c00964)
Supplement: Supplementary file 1 [file ao6c00964_si_001.pdf]

A Smartphone-Assisted Approach to Formaldehyde Detection using Diethanolamine-Grafted Carbon Nanoparticles

Rossella Santonocito[1], Lorenzo Russo[1], Victor Sebastian[2,3,4,5], Angelo Ferlazzo[1], Antonino Gulino[1], Manuel Petroselli[6], Roberta Ruffino[1,7], Giovanni Li Destri[1,7], Andrea Pappalardo[1], Nunzio Tuccitto[1,4], Alessia Cavallaro[1]*, Giuseppe Trusso Sfrazzetto[1]*

[1] Department of Chemical Sciences, University of Catania, viale A. Doria 6, 95125, Catania, Italy.

[2] Instituto de Nanociencia y Materiales de Aragoń Aragón (INMA), CSIC-Universidad de Zaragoza, Campus Rio Ebro, Edificio I + D + I, C/Poeta Mariano Esquillor, s/n, 50018, Zaragoza, Spain

[3] Department of Chemical and Environmental Engineering, Institute of Nanoscience and Materials of Aragon, Universidad de Zaragoza, Zaragoza, Spain

[4] Networking Research Center in Biomaterials, Bioengineering and Nanomedicine (CIBER-BBN), Instituto de Salud Carlos III, 28029, Madrid, Spain

[5] Laboratorio de Microscopías Avanzadas, Univ. de Zaragoza, 50018, Zaragoza, Spain

[6] Department of Science and Technological Innovation, University of Eastern Piedmont "Amedeo Avogadro", Viale Teresa Michel 11, 15121, Alessandria, Italy

[7] CSGI Consorzio Interuniversitario per lo sviluppo dei Sistemi a Grande Interfase, Via della Lastruccia 3, Firenze, Italy

Corresponding authors:

Giuseppe Trusso Sfrazzetto – giuseppe.trusso@unict.it

Alessia Cavallaro – alessia.cavallaro@phd.unict.it

Supporting Information

1. Structural characterization

1.1. XPS

Table S1. XPS Binding Energies and assignments.

Atomic state	B. E. (eV)	Assignment
**C 1s**	284.5	sp^2
	285.0	sp^3 C-C, C-H
	285.8	C-N
	286.3	C-OH
	288.2	O=C-N
**O 1s**	531.8	O=C-N
	533.3	C-OH
	533.8	O-Si
**N 1s**	399.8	O=C-N
	401.6	N^+

1.2. ^{1}H NMR

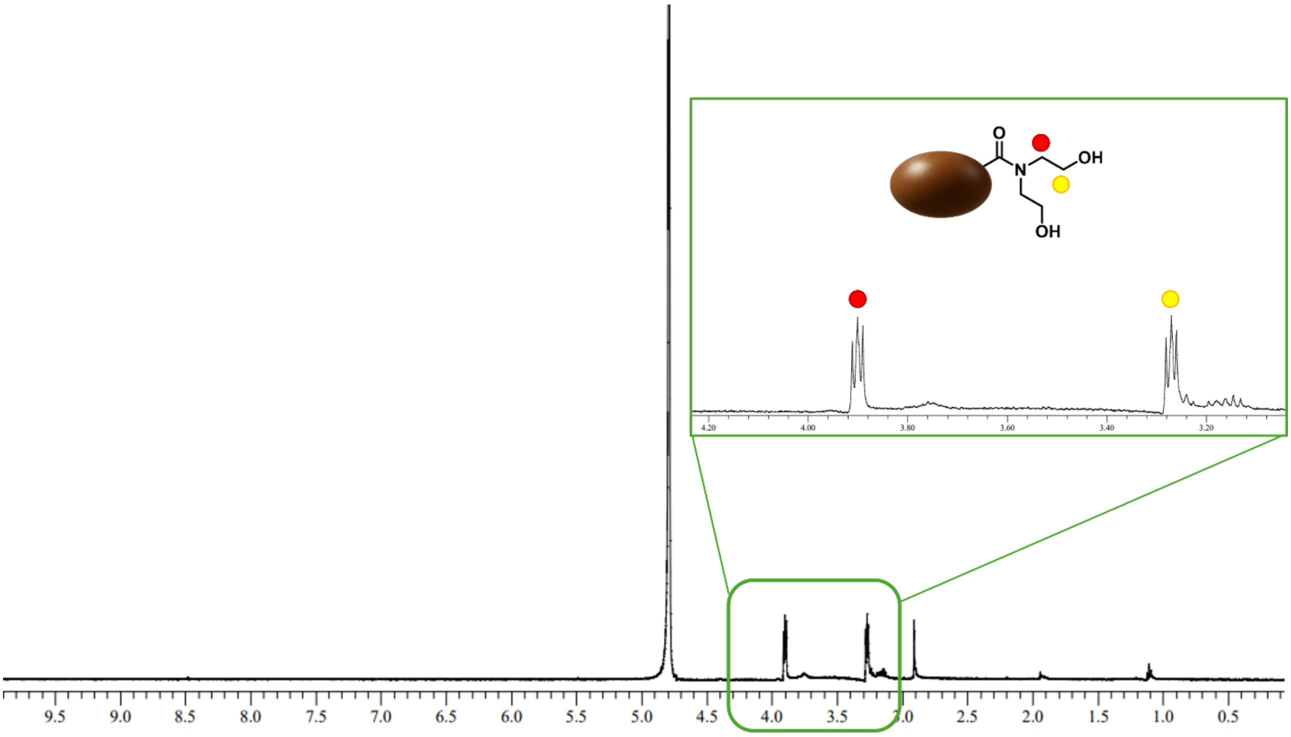

Figure S1. ^{1}H NMR spectrum of CNPs-DEA in D_2O. The inset shows the aliphatic region between 3.1 and 4.2 ppm.

1.3. FT-IR

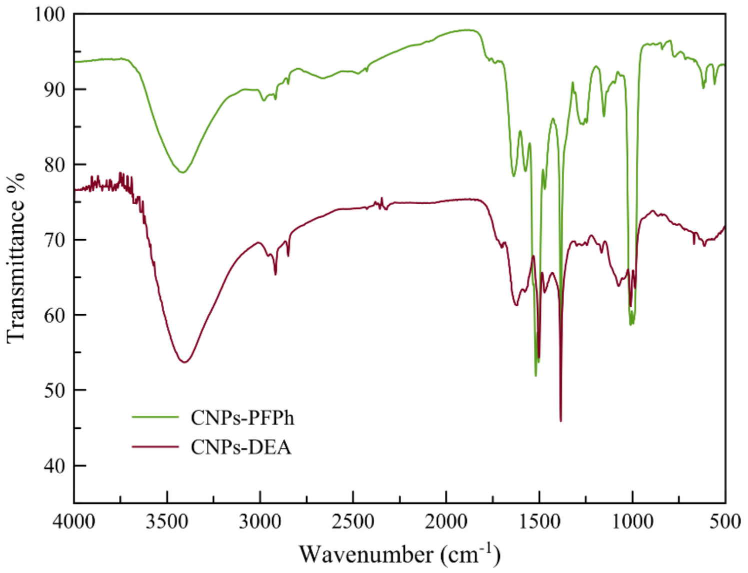

Figure S2. Comparison of FT-IR spectra of CNPs-PFPh (in green) and CNPs-DEA (in red).

2. Calculation of CNPs-DEA molar concentration

To determine the molar concentration of CNPs-DEA and subsequently calculate the apparent binding constant, we combined theoretical estimations with experimental data. Based on AFM measurements, the dimensions of the CNPs were used to estimate their surface area and, consequently, the maximum number of diethanolamine molecules that could be accommodated in the functionalization shell. To estimate the number of carbon atoms per nanoparticle, we considered literature data[1] and approximated the nanoparticle structure as a stack of graphene sheets. Using the average interlayer distance in graphene and the known carbon atom density per unit area, we calculated the approximate number of carbon atoms in a single nanoparticle and derived its molecular weight.

Finally, knowing the mass concentration of CNPs-DEA in solution, we converted it to molar concentration. This approach gave a value of approximately 1×10^{-8} M, which was used as host concentration in HypSpec for the determination of the binding constant.

3. HypSpec output

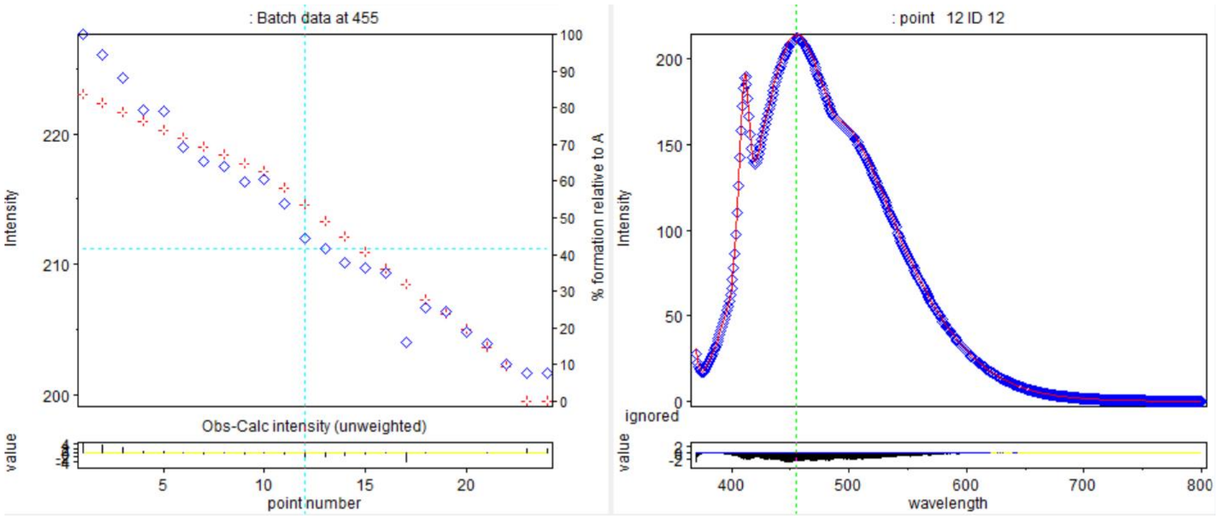

Figure S3. HypSpec output file for fluorescence titration.

```
Converged in 1 iteration with sigma = 1,0361

                      standard
Log beta    value     deviation
AB          4.3072    0.0021
```

4. Selectivity tests

For each interferent, aqueous stock solutions were prepared at a concentration of 10^{-2} M. The water solubility of each interferent is reported in **Table S2**. A fixed volume of 2 μL from an interferent stock solution was added to a cuvette, containing a final volume of 2 mL of receptor + interferent in MilliQ water. This results in an interferent concentration of 10 ppm. The same procedure was repeated for each interferent.

Table S2. Solubility values of each interferent in water at 25°C, according to literature.

Interferent	Solubility (g L^{-1})	Ref.
Acetaldehyde	miscible in all proportions	2
Propionaldehyde	306	
iso-butyraldehyde	56.2	

Benzaldehyde	6.95	3
Methanol	miscible in all proportions	4
Acetone	miscible	5
Ethyl acetate	78.9	6
Toluene	0.556	7

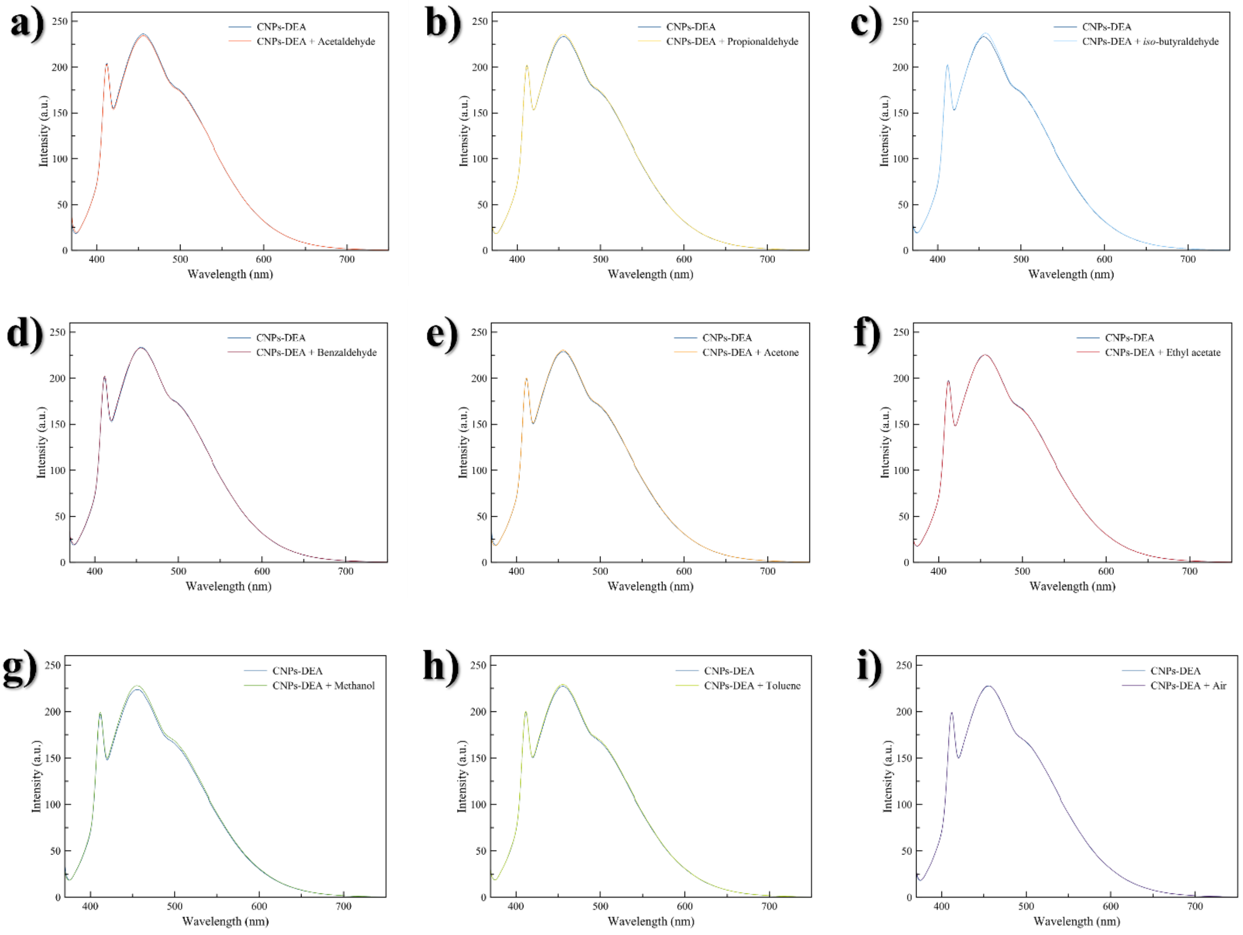

Figure S4. Overlap of CNPs-DEA emission spectrum and CNPs-DEA + interferent (10 ppm) emission spectrum: a) acetaldehyde, b) propionaldehyde, c) *iso*-butyraldehyde, d) benzaldehyde, e) acetone, f) ethyl acetate, g) methanol, h) toluene and i) air.

5. Computational analysis of the sensing mechanism

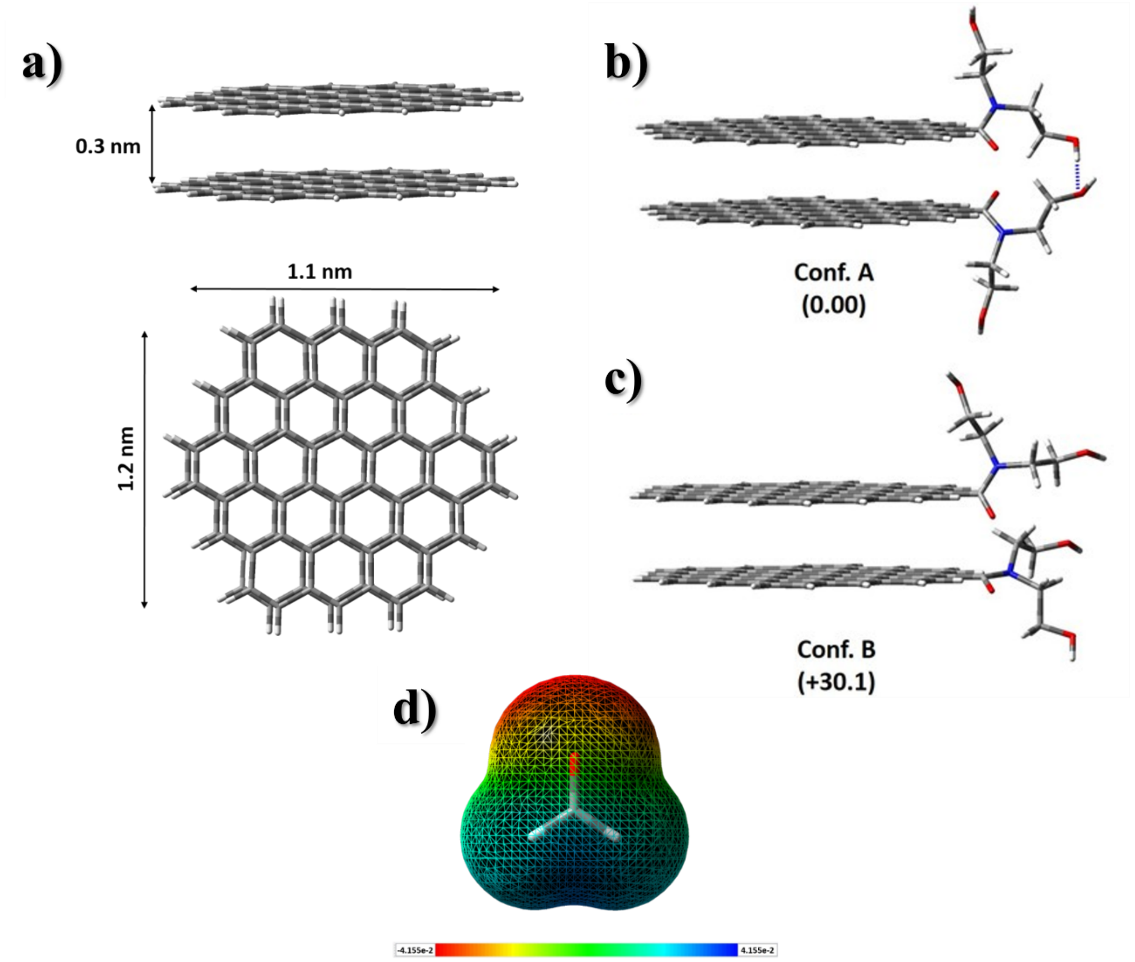

Figure S5. a) Schematic representation of the unfunctionalized graphene bilayer. Length, size and interspace between the two layers are reported in nm. b-c) Conformational screening on the CNPs-DEA model system at DFT level of theory, taking into account the XPS data experimentally observed. Relative energies (ΔE) are reported between parentheses and expressed in kcal/mol, while hydrogen bonds are marked with dash blue lines. d) Molecular electrostatic potential (MEP), calculated at DFT level of theory, of the formaldehyde guest.

Table S3: Complexation energy ($E_{complex}$) for FA@CNPs-DEA complex calculated at B3LYP/6-31G(d,p) level of theory in the gas phase. Complexation energy of a water dimer ($2H_2O$) is reported for a better comparison. Energy difference (ΔE) is reported, taking into account the FA-CNPs-DEA complex as a reference.

HG Complex	$E_{complex}$ (kcal/mol)	ΔE (kcal/mol)
**FA-CNPs-DEA**	10.7	-

**$2H_2O$ (dimer)**	5.9	- 4.8

6. Gaseous FA detection by test strips

6.1 Assumptions for the generation of accurate gaseous FA concentration

The generation of controlled gaseous FA concentrations was calculated based on the liquid-vapor equilibrium of aqueous FA solutions. Due to non-ideal behavior that precludes the use of Henry's law, the empirical correlation established by Dong and Dasgupta was utilized.[8] This model is applicable within the concentration range 1.00×10^{-6} M to 6.86×10^{-3} M. The empirical equation is the following (1):

$$[HCHO_{aq}] = 10^{x}[HCHO_{g}]^{y} \quad (1)$$

At 25 °C, the empirical constants are *x* = 3.8865 and *y* = 1.065. The model indicates that parameters such as pH and salinity do not influence the equilibrium. The conversion from partial pressure (atm) to concentration in ppm was performed assuming $P_{atm} = 1$, where partial pressure is equivalent to the molar ratio (χ_{HCHO}), according to equation (2). The equation also considers the conversion to ppm:

$$HCHO\ (ppm) = \frac{P_{HCHO}}{P_{atm}} \cdot 10^{6} \cong P_{HCHO} \cdot 10^{6} \quad (2)$$

These mathematical relationships were used to determine the precise aqueous FA concentrations required to achieve the desired gas-phase levels.

6.2. Sensing procedure

Fluorescence readout was performed using a custom-engineered 3D-printed dark chamber equipped with a 365 nm UV-LED excitation source. Images were acquired using a commercial smartphone featuring a 12-megapixel camera sensor. The chamber design ensured a fixed working distance of 20 cm between the camera and the sensor, eliminating ambient light interference. For the gas-phase experiments, the sensing strips were attached to the inner face of 20 mL vial caps. The vials were subsequently sealed and maintained under static conditions in a temperature-controlled room at 25 °C. Upon reaching equilibrium, as determined by the kinetic studies, the vials were unsealed, and the sensors were immediately imaged under UV LED irradiation. The captured files were then processed to extract the relevant analytical data.

6.3. Image elaboration

To extract accurate analytical data, the raw images underwent a two-step processing protocol. First, background noise was removed using Origin by applying a threshold mask that zeroed the intensity of non-sensing regions (background = 0), thereby isolating the active spots. The images were then imported into ImageJ (**Figure S7**) for intensity quantification. The RGB color data were transformed into a single grayscale value (G), by the application of the following formula: G = (R_{value} + G_{value} + B_{value})/3. Finally, the sensing performance was evaluated by determining the normalized emission intensity (GI_n). This metric relates the signal measured after FA exposure (GI_{sample}) to the initial control signal (GI_{blank}), according to:

$$GI_n = \frac{GI_{sample}}{GI_{blank}}$$

With:

- GI_n = normalized fluorescence intensity of the sensor
- GI_{sample} = fluorescence intensity of the sensor after exposure to gaseous FA
- GI_{blank} = fluorescence intensity of the sensor before any exposure.

The whole procedure described was applied to both kinetic and calibration experiments.

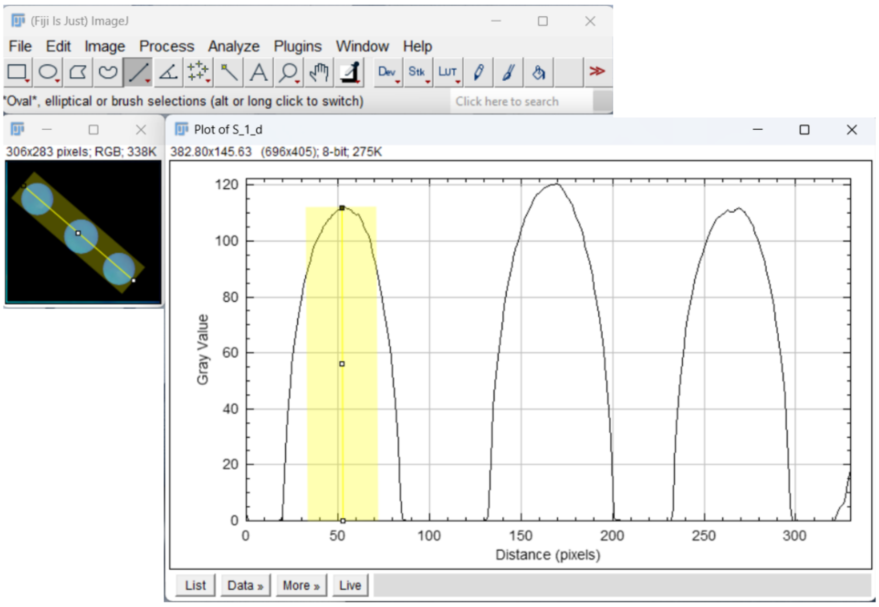

Figure S6. Extrapolation of the normalized intensity from image using the program ImageJ.

7. Analysis of a real sample

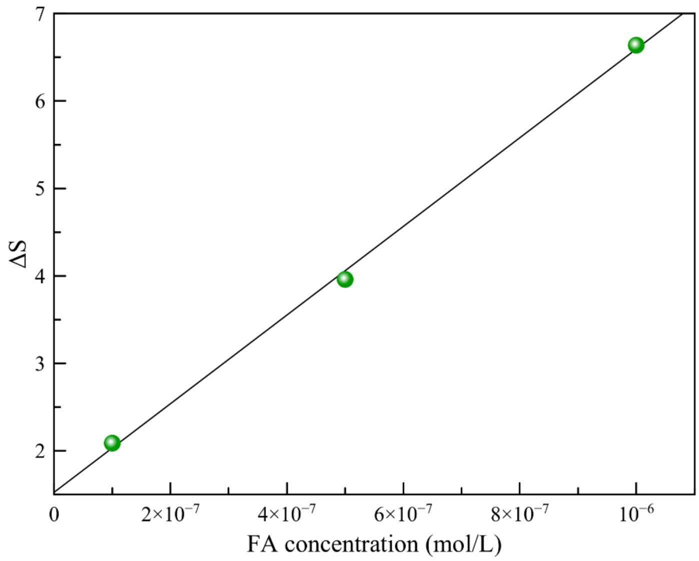

Figure S7. FA quantification in paint sample: standard additions of 2, 10 and 20 μL of 10^{-2} M FA solution in MilliQ water, in the same experimental conditions as those reported for fluorescence titration.

References

1. Fuchs, H.-N.; Goerbig, M. O. Introduction to the Physical Properties of Graphene, **2008**. https://web.physics.ucsb.edu/~phys123B/w2015/pdf_CoursGraphene2008.pdf.

2. Smith, T. E.; Bonner, R. F. Acetaldehyde, Propionaldehyde, and n-Butyraldehyde. *Ind. Eng. Chem.* **1951**, *43*, 1169-1173. https://doi.org/10.1021/ie50497a049.

3. Stephenson, R. M. Mutual solubility of water and aldehydes. *J. Chem. Eng. Data* **1993**, *38*, 630-633. https://doi.org/10.1021/je00012a040.

4. Sun, M.; Wang, L.-M.; Tian, Y.; Liu, R.; Ngai K. L.; Tan, C. Component Dynamics in Miscible Mixtures of Water and Methanol. *J. Phys. Chem. B* **2011**, *115*, 8242-8248. https://doi.org/10.1021/jp202893v.

5. Haynes, W. M. CRC Handbook of Chemistry and Physics, 97th ed., CRC Press in Taylor & Francis Group: Boca Raton, FL, **2017**. ISBN 9781498754286.

6. Segatin, N.; Klofutar, C. Thermodynamics of Solution of Some Alkyl Acetates in Water. *Monatshefte für Chemie / Chemical Monthly* **2001**, *132*, 1451-1462. https://doi.org/10.1007/s007060170002.

7. Dohányosová, P.; Fenclová, D.; Vrbka, P.; Dohnal, V. Measurement of Aqueous Solubility of Hydrophobic Volatile Organic Compounds by Solute Vapor Absorption Technique: Toluene, Ethylbenzene, Propylbenzene, and Butylbenzene at Temperatures from 273 K to 328 K. *J. Chem. Eng. Data* **2001**, *46*, 1533-1539. https://doi.org/10.1021/je010172r.

8. Dong, S.; Dasgupta, P. K. Solubility of gaseous formaldehyde in liquid water and generation of trace standard gaseous formaldehyde. *Environ. Sci. Technol.* **1986**, *20*, 637-640. https://doi.org/10.1021/es00148a016.
